# Brain IGF-1 Receptors Control Mammalian Growth and Lifespan through a Neuroendocrine Mechanism

**DOI:** 10.1371/journal.pbio.0060254

**Published:** 2008-10-28

**Authors:** Laurent Kappeler, Carlos De Magalhaes Filho, Joëlle Dupont, Patricia Leneuve, Pascale Cervera, Laurence Périn, Catherine Loudes, Annick Blaise, Rüdiger Klein, Jacques Epelbaum, Yves Le Bouc, Martin Holzenberger

**Affiliations:** 1 INSERM U893, Hôpital Saint-Antoine, Paris, France; 2 Université Pierre-et-Marie-Curie, Paris, France; 3 INRA, Nouzilly, France; 4 Service d'Anatomopathologie, Hôpital Saint-Antoine, Paris, France; 5 INSERM U549, Centre Paul Broca, Paris, France; 6 Department of Molecular Neurobiology, Max-Planck Institute of Neurobiology, Munich-Martinsried, Germany; The Salk Institute, United States of America

## Abstract

Mutations that decrease insulin-like growth factor (IGF) and growth hormone signaling limit body size and prolong lifespan in mice. In vertebrates, these somatotropic hormones are controlled by the neuroendocrine brain. Hormone-like regulations discovered in nematodes and flies suggest that IGF signals in the nervous system can determine lifespan, but it is unknown whether this applies to higher organisms. Using conditional mutagenesis in the mouse, we show that brain IGF receptors (IGF-1R) efficiently regulate somatotropic development. Partial inactivation of IGF-1R in the embryonic brain selectively inhibited GH and IGF-I pathways after birth. This caused growth retardation, smaller adult size, and metabolic alterations, and led to delayed mortality and longer mean lifespan. Thus, early changes in neuroendocrine development can durably modify the life trajectory in mammals. The underlying mechanism appears to be an adaptive plasticity of somatotropic functions allowing individuals to decelerate growth and preserve resources, and thereby improve fitness in challenging environments. Our results also suggest that tonic somatotropic signaling entails the risk of shortened lifespan.

## Author Summary

Using a mouse model relevant for humans, we showed that lifespan can be significantly extended by reducing the signaling selectively of a protein called IGF-I in the central nervous system. This effect occurred through changes in specific neuroendocrine pathways. Dissecting the pathophysiological mechanism, we discovered that IGF receptors in the mammalian brain efficiently steered the development of the somatotropic axis, which in turn affected the individual growth trajectory and lifespan. Our work confirms experimentally that continuously low IGF-I and low growth hormone levels favor extended lifespan and postpone age-related mortality. Together with other recent reports, our results further challenge the view that administration of GH can prevent, or even counteract human aging. This knowledge is important since growth hormone is often prescribed to elderly people in an attempt to compensate the unwanted effects of aging. Growth hormone and IGF-I are also substances frequently used for doping in sports.

## Abstract 3

Inactivating IGF receptors in the brain decreased growth hormone and IGF-I, and increased lifespan in healthy mice. Such neuroendocrine longevity could be a physiological response to environment.

## Introduction

Growth hormone (GH) and insulin-like growth factors (IGFs) promote mammalian growth [1,2] and contribute to metabolic regulation [3–5]. In contrast, inhibiting the actions of GH or IGF extends lifespan: mice constitutively lacking growth hormone receptor (GHR knockout [6,7]) or GH-releasing hormone receptor (GHRHR^lit/lit^ mutant [8,9]), or mice with fewer IGF receptors [10], no insulin-receptor substrate 1 (IRS1) [11], or diminished IRS2 [12] live longer. This powerful effect of insulin-like signals on longevity was discovered in nematodes [13,14] and insects [15,16], but may extend to humans: functionally relevant IGF receptor (IGF-1R) mutations have recently been discovered in centenarians [17], and conditions of low IGF-I, PI3K, IRS1, GH, and GHRH correlate with prolonged lifespan [18,19]. Significantly, the regulation of aging in diverse species involves common major hormonal pathways, and the underlying mechanisms have clearly been conserved through evolution. Several lines of evidence suggest that the fundamental biological process might be an adaptive mechanism enabling individuals to adjust body size, metabolism, and lifespan to their environment, in particular to the challenging natural fluctuations of resources [20–22]. Such an adaptive mechanism would require individual plasticity, especially of the somatotropic hormone axis in response to environmental cues. Because the development of this hormone axis is steered by the central nervous system (CNS) through hypothalamic control of pituitary differentiation [23], it is possible that any somatotropic plasticity would involve the CNS. Indeed, previous work in nematodes and insects showed that insulin-like signals in the nervous system alter survival in a non-cell autonomous manner [24–29], suggesting that neuronal control of aging through insulin-like signals might also be conserved. To test these ideas experimentally in a mammalian model, we genetically manipulated IGF signaling in the mouse brain and explored growth, metabolism, and lifespan in these mutants.

We found that brain IGF receptors strongly promote the development of the somatotropic function in mice. We show that developmental IGF signaling in the brain selectively determines somatotropic plasticity, regulates GH and IGF-I secretion, and thereby controls growth of peripheral tissues, adult glucose metabolism, and energy storage, as well as survival and mortality.

## Results

### Brain IGF-1R Regulates Growth Hormone

**Figure 1:**
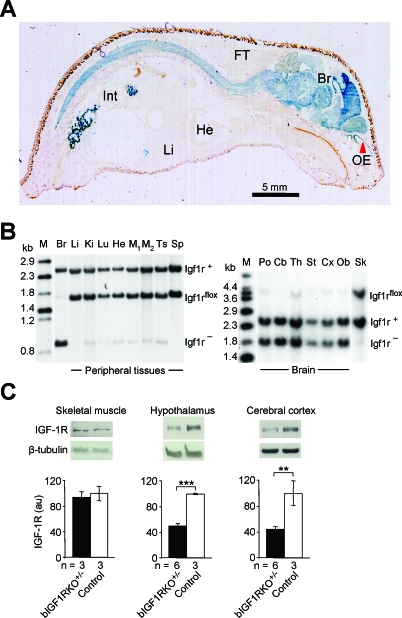
Brain-Targeted Inactivation of the *Igf1r* Gene Using Cre-lox Mutagenesis (A) CNS-specific Cre-lox recombination demonstrated using X-Gal staining (blue) in a sagittal section from a 2-wk-old *NesCre* ^+/0^ mouse harboring a LacZ reporter (*Rosa26R* ^+/0^) [33]. *NesCre* is expressed in neuroepithelium by neuronal and glial precursors. Abbreviations: Br, brain; FT, fat tissue; He, heart; Int, intestine (with bacterial artifacts); Li, liver; OE, olfactory epithelium (red arrow). (B) Southern blot analysis of adult bIGF1RKO ^+/−^ tissues revealed complete recombination in the brain (Br) and the intact *Igf1r^flox^* allele in all peripheral tissues (left panel). Recombination in peripheral tissues was minimal. The IGF-1R knockout was effective throughout the brain (right panel) and stable through time (unpublished data). The restriction enzymes used were HincII and I-SceI (left blot) and HincII alone (right). Cb, cerebellum, Cx, cortex, Ki, kidney, Lu, lung, M, DNA size marker, M1/M2, skeletal muscle, Ob, olfactory bulb, Po, pons, Sk, skin, Sp, spleen, St, striatum, Th, thalamus, Ts, testis. (C) bIGF1RKO ^+/−^ mice had normal IGF-1R levels in peripheral tissues (e.g., muscle) and ∼50% of normal levels in the CNS (here: hypothalamus and cortex), as assessed by western blotting.

**Figure 2:**
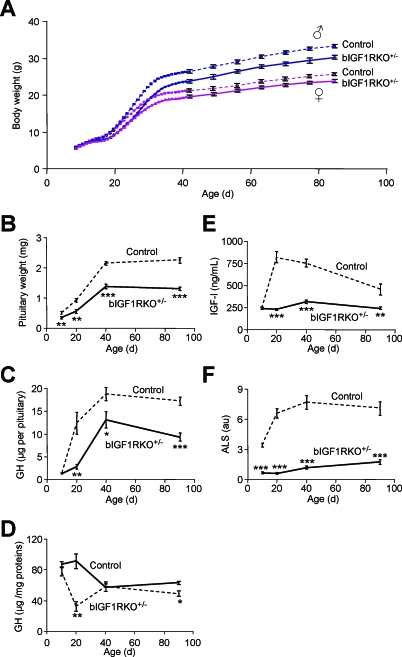
Growth and Postnatal Development of the Somatotropic Axis in Mutant and Control Mice (A) bIGF1RKO^+/−^ mice had significantly delayed growth, from age 18 d onwards. (B) bIGF1RKO^+/−^ pituitaries were small from age 10 d onwards (*n* = 10 per group). (C) Pituitaries from mutants contained little GH (*n* = 5 per group). (D) Data from (C) expressed per milligram of pituitary protein revealing a selective drop at age 20 d. (E) In control mice, serum IGF-I increased rapidly after age 10 d (in response to endogenous GH), but remained low in bIGF1RKO^+/−^ mice. (F) Similar to IGF-I (E), the postnatal surge of ALS in controls was absent from bIGF1RKO^+/−^ mice (*n* = 5 per group).

**Figure 3:**
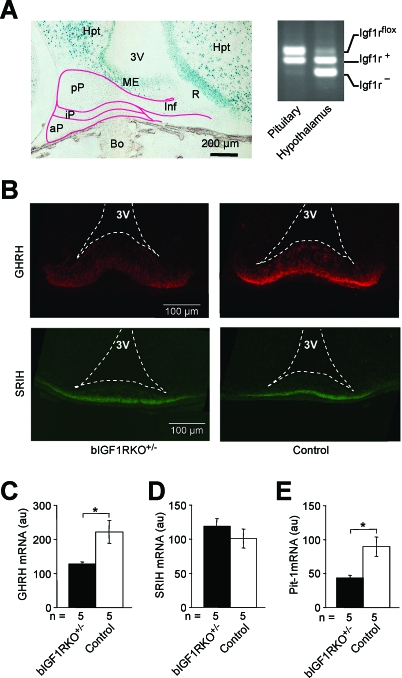
Somatotropic Signals in the Hypothalamic-Pituitary Complex (A) Efficient Cre-lox recombination in hypothalamus, not in pituitary, as shown by X-Gal-staining of a sagittal *NesCre* ^+/0^ *;Rosa26R* ^+/0^ brain section: the anterior pituitary originates from the pharyngeal wall, not from neuroepithelium, to which *NesCre* transgene expression is confined. Abbreviations: 3V, third ventricle; aP, anterior pituitary; Bo, bone; Hpt, hypothalamus; Inf, infundibulum; iP, intermediate pituitary; ME, median eminence; pP, posterior pituitary; R, recessus of the third ventricle. PCR analysis of genomic DNA confirmed absence of recombination from the pituitary: intact *Igf1r* ^flox^ alleles in pituitary, and knockout (*Igf1r* ^−^) alleles prevalent in hypothalamus. (B) GHRH immunoreactivity (red) at nerve endings in the ME was weaker than control in bIGF1RKO^+/−^ mice (65 ± 6 versus 122 ± 10 au (arbitrary units), *p* < 0.01, *n* = 3), whereas SRIH (green) was unaffected (191 ± 3 versus 197 ± 5 au, *n* = 3). Briefly, ME tissue sections from the same anatomical location in three bIGF1RKO^+/−^ and three control 10-day-old males were subjected to IHC. Micrographs were taken under identical conditions. For each animal, data from ten sections were averaged. The ratio of GHRH to SRIH immunoreactivity was lower in bIGF1RKO^+/−^ than in control animals (0.34 ± 0.05 versus 0.54 ± 0.09, *n* = 6; *p* = 0.06). (C) Similarly, GHRH gene expression (measured by quantitative real-time RT-PCR, relative to β-actin) was lower at age 10 d in mutants than controls. (D) SRIH expression was similar in mutants and controls. (E) Pit-1 expression (relative to 18S rRNA) was lower in bIGF1RKO ^+/−^ than wild-type pituitaries at age 10 d.

To study the role of IGF signaling in the CNS, we generated mice with heterozygous and homozygous brain-specific IGF-1 receptor knockout mutations (bIGF1RKO ^+/−^ and bIGF1RKO ^−/−^) by conditional mutagenesis (Figure 1A and 1B). Mutants and their controls were littermates with identical genetic background, and matings were performed such that the Nestin-Cre transgene was always paternally transmitted (see Methods and Text S1 for breeding and genetic background). Homozygous mutants (bIGF1RKO ^−/−^) have no IGF-1R on CNS neurons or glia. They were microcephalic and developed a complex phenotype involving severe growth retardation, infertility, and abnormal behavior (Figure S1). Though very interesting per se, the homozygous bIGF1RKO^−/−^ mice did not show extended lifespan and their adult plasma IGF-I concentration was significantly higher than control values (Figure S1E and S1G). Thus, homozygous mutants were not a suitable model for healthy longevity, which is generally associated with diminished insulin-like signaling [20,21]. We therefore studied the heterozygous mutants (bIGF1RKO ^+/−^), in which the IGF-1R levels in the CNS are half that in the wild-type (Figure 1C). They were healthy and behaved normally (Figure S2). Their body growth, however, though initially normal, was progressively retarded from 20 d of age onwards (Figure 2A). By age 90 d, bIGF1RKO ^+/−^ adults weighed about 90% of controls (males, 30.5 ± 0.6 g, *n* = 12 versus 33.7 ± 0.4 g, −9.6%, *n* = 19, *p* < 0.0001; females, 24.1 ± 0.3 g, *n* = 18 versus 26.2 ± 0.5 g, −7.9%, *n* = 14, *p* < 0.001) and were 5% shorter than controls (*p* < 0.001) (Table S1). bIGF1RKO ^+/−^ mice had normal IGF-1R levels in peripheral tissues (see Figure 1C), so we speculated that endocrine growth regulation during development was disturbed. bIGF1RKO ^+/−^ pituitaries were indeed small from age 10 d onwards (Figure 2B), and total GH content remained low throughout development (Figure 2C). The GH concentration per milligram protein fell at age 20 d, suggesting that retardation of early postnatal somatotroph differentiation (Figure 2D) started between day 10 and day 20. Plasma IGF-I, which strongly depends on GH, did not show any pubertal increase in bIGF1RKO ^+/−^ mice while controls displayed the normal surge (Figure 2E). Moreover, the concentration of the acid labile subunit (ALS), an important regulator of IGF-I stability and itself regulated by GH, was very low in mutants throughout postnatal life (Figure 2F). Importantly, in this model the IGF-1R gene is knocked out in the hypothalamus but not in the pituitary (Figure 3A). Therefore, we suspected that this somatotropic phenotype was caused by alterations in GH-regulatory neurons of the hypothalamus, i.e. arcuate nucleus GHRH neurons and anterior periventricular somatostatin (SRIH) neurons whose endings converge on the external layer of the median eminence (ME). Indeed, hypothalamic GHRH expression in bIGF1RKO ^+/−^ mice was significantly low, and GHRH accumulation in the GHRH neuron endings was clearly diminished around age 10 d (Figure 3B and 3C). In contrast, hypophysiotropic SRIH-producing neurons in the hypothalamus exhibited a normal abundance of SRIH at age 10 d, evidence of the cell-specificity of this phenotype (Figure 3B and 3D). Accordingly, Pit-1 expression, which is controlled by GHRH neurons and steers somatotropic cell differentiation, was half normal in mutant pituitaries (Figure 3E).

**Figure 4:**
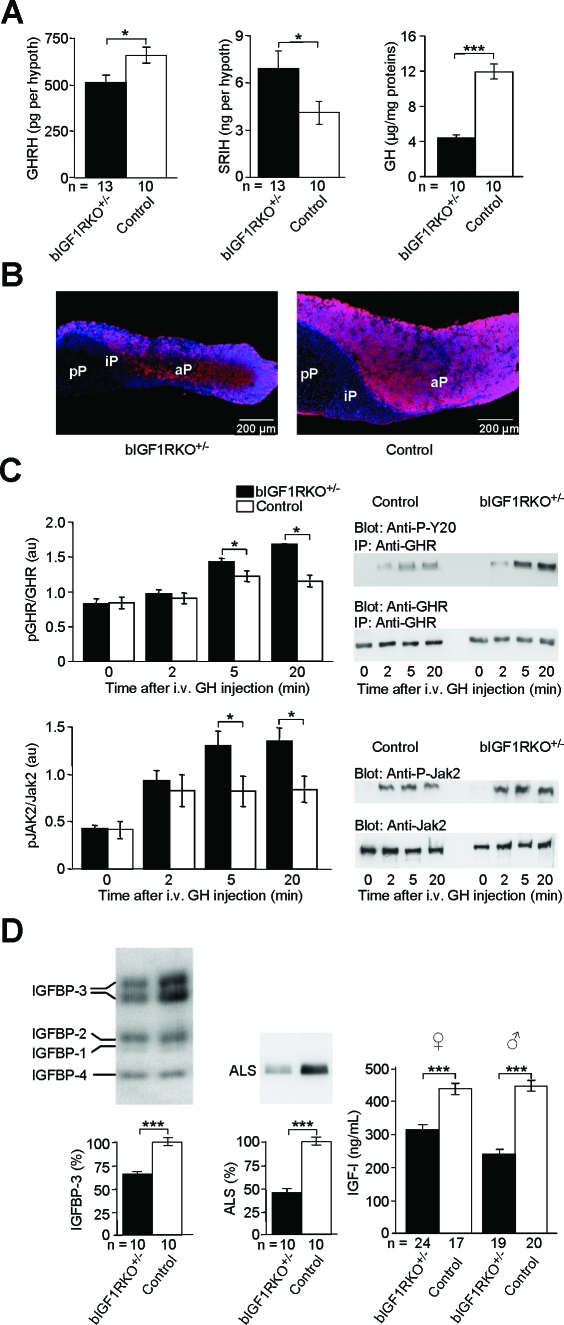
GH/IGF-I Deficiency in Adult bIGF1RKO^+/−^ (A) Hypothalamic GHRH was higher (left) and SRIH lower (middle) in bIGF1RKO^+/−^ than control mice at age 9 mo. Pituitary GH was markedly low (right). The wet weight of the hypothalamus and total proteins extracted from hypothalamus in mutants did not differ from control values (bIGF1RKO^+/−^ 1.55 ± 0.12 mg versus controls 1.53 ± 0.10 mg, *p* = 0.86, NS). (B) GH immunoreactivity (red) was much lower in bIGF1RKO^+/−^ than control pituitaries, here in frontal sections through the lateral half gland with DAPI (blue) nuclear counter-stain. aP, anterior pituitary; iP, intermediate pituitary; pP, posterior pituitary. (C) Liver GH receptor (upper) and Jak2 (lower panel) were overphosphorylated upon administration of GH into the portal vein, indicating hypersensitivity of GHR (*n* = 4 per group; paired *t*-test). Right: representative western blots. (D) IGF-binding protein-3 (IGFBP-3, left), ALS (middle), and circulating IGF-I (right) were significantly less abundant in bIGF1RKO^+/−^ mice than controls, indicative of deficient peripheral GH action. IGFBP-3 and ALS (and also Spi2.1, see text) were determined only in males.

This early somatotropic deficiency persisted: in adult bIGF1RKO ^+/−^ hypothalamus, the GHRH concentration remained low, while the abundance of antagonistic SRIH increased (Figure 4A). Consistent with these observations, adult bIGF1RKO ^+/−^ pituitaries were small (males, −40%; females, −33%; *p* < 0.001; Table S1), contained many fewer GH-producing cells, and were GH depleted compared with controls (Figure 4A right, 4B). Peripheral tissues showed diverse evidence of chronic lack of GH stimulation: Liver GH receptors were hypersensitive to GH stimulation (Figure 4C), and several markers of peripheral GH action were abnormally low in adults (IGFBP-3, −37%; ALS, −53%; Figure 4D; serine protease inhibitor Spi2.1, −53%, unpublished data). As expected, plasma IGF-I levels were lower in adult bIGF1RKO ^+/−^ mice than controls (males, 243 ± 10 versus 453 ± 15 ng/ml, −46%, *p* < 0.0001; females, 321 ± 13 versus 442 ± 16 ng/ml, −27%, *p* < 0.0001) (Figure 4D, right). Finally, IGF-1R was consistently underphosphorylated in major peripheral tissues (e.g. skeletal muscle: between 51% and 67% of control levels, depending on age, as determined by IGF-1R immunoprecipitation and western immunoblot; Figure S3), clearly indicating that cells were receiving less than wild-type IGF-I stimulation.

### Gonado- and Thyrotropic Functions Are Preserved

To determine whether the observed hypothalamic inhibition of endocrine GH and IGF-I was specific, we assessed gonado- and thyrotropic functions in mutant animals by monitoring fertility, reproduction, and energy metabolism. bIGF1RKO^+/−^ females were fertile and had normal litters (7.1 ± 0.7 pups, *n* = 6 litters). Ovarian cycling, controlled by anterior pituitary follicle-stimulating hormone (FSH), luteinizing hormone (LH), and their hypothalamic releasing hormones, was identical in bIGF1RKO^+/−^ and control females (mean estrous cycle length, at 4 mo of age, 5.1 ± 0.3 versus 4.9 ± 0.3 d, *n* = 15, not significant [NS]; at ∼9 mo of age, 5.3 ± 0.2 versus 5.9 ± 0.4 d, *n* = 37, NS). Moreover, there was no difference in pro-estrus estradiol concentration in adult females (82.3 ± 6.3 versus 85.2 ± 5.0 pmol/l, *n* = 39, NS) nor in ovary size relative to weight (bIGF1RKO^+/−^ 0.36 ± 0.01 versus controls 0.38 ± 0.02 mg/g, *n* = 72, NS); therefore we considered gonadotropic function to be normal. Plasma thyroxin (T4) levels, controlled by pituitary thyroid-stimulating hormone (TSH) were unaffected in females (bIGF1RKO^+/−^ 29.1 ± 1.4, *n* = 19 versus controls 27.5 ± 1.6 μg/l, *n* = 12, NS) and, although they were moderately high in males (bIGF1RKO^+/−^ 39.0 ± 2.0, *n* = 14 versus controls 30.0 ± 1.0 μg/l, *n* = 20, *p* < 0.005), we observed no differences in core body temperature (males, bIGF1RKO^+/−^ 36.7 ± 0.3 °C versus controls 37.0 ± 0.4 °C, *n* = 15, NS; females, bIGF1RKO^+/−^ 37.4 ± 0.1 °C versus controls 37.5 ± 0.1 °C, *n* = 21, NS), nor did we find abnormalities in physical activity or food consumption (Figure S2A and S2B). Hence, we concluded that thyrotropic function also was in the normal range. In addition, the development of gonado- and thyrotropic phenotypes between 4 and 9 mo of age did not suggest that these functions differed thereafter.

### Energy Storage Is Altered in bIGF1RKO^+/−^ Mice

**Figure 5:**
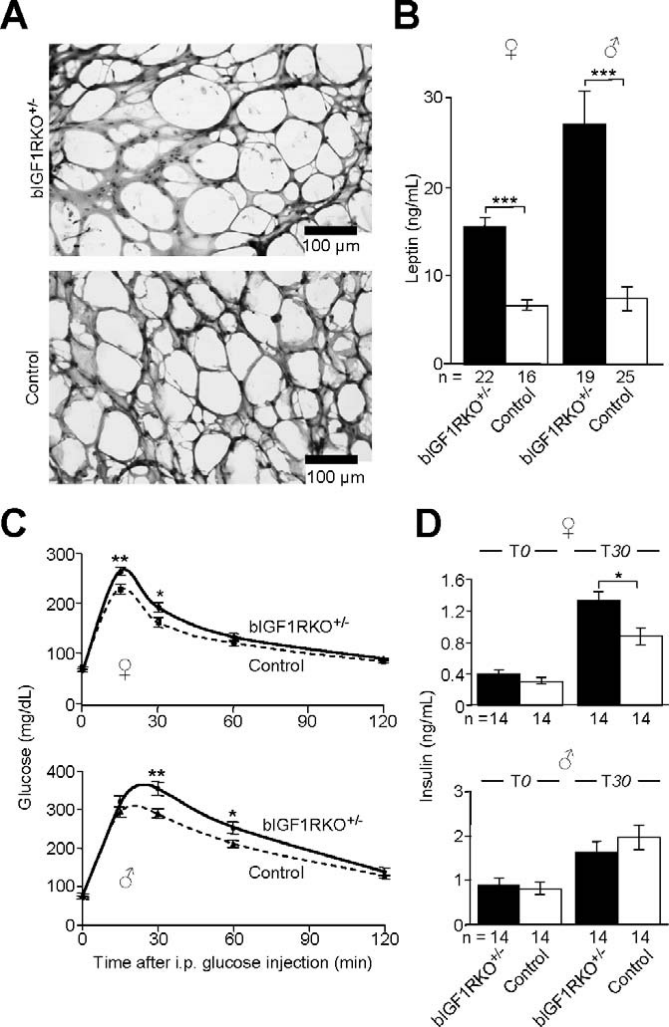
Exploration of Adult Energy Metabolism (A) bIGF1RKO^+/−^ adipocytes from inguinal AT were abnormally enlarged. (B) Leptinemia was very high in bIGF1RKO^+/−^ mice, and strongly correlated with individual total AT size in all animals (*R* = 0.76, *p* < 0.0001, *n* = 82). (C) Glucose tolerance was impaired in bIGF1RKO^+/−^ males and females. (D) 30 min after intraperitoneal glucose injection (T*30*), the plasma insulin concentration was, as expected, significantly increased in mutants and controls. However, although bIGF1RKO^+/−^ females responded to elevated blood glucose with adequate hyperinsulinemia, bIGF1RKO^+/−^ males did not, suggesting a secretory defect.

Weight gain with age was slightly greater for adult bIGF1RKO^+/−^ mice than for controls (unpublished data), such that female mutants finally attained the same weight as controls. We analyzed body composition in 10-mo-old animals, and as suggested by growth curves and circulating IGF-I levels, most organs in bIGF1RKO ^+/−^ mice were smaller than those in controls (Table S1). Adipose tissue (AT), in contrast, was significantly enlarged in males and females (Figure 5A; Table S2). In both sexes, the largest increase was in subcutaneous AT, whereas visceral AT was less increased in mutant females, and even slightly diminished in males, similar to other GH-deficient mouse models [30–32]. Accordingly, circulating leptin levels were excessive in bIGF1RKO^+/−^ mice (males, 27.1 ± 3.6 ng/ml versus 7.4 ± 1.2 ng/ml; females, 15.5 ± 1.0 ng/ml versus 6.5 ± 0.5 ng/ml; both *p* < 0.0001; Figure 5B). At age 4 mo, blood biochemistry in bIGF1RKO^+/−^ mice was still normal (Table S3), but at 10 mo, HDL and total cholesterol concentrations were high (unpublished data) and triglyceride (TG) and free fatty acid (FFA) concentrations in males significantly higher than in controls, probably due to the abundance of AT (TG, 1.36 ± 0.12 mmol/l versus 1.03 ± 0.09 mmol/l, *n* = 10 per group, *p* < 0.05; FFA, 0.65 ± 0.04 mmol/l versus 0.48 ± 0.03 mmol/l, *n* = 10 per group, *p* < 0.005). As circulating GH counteracts fat storage, these metabolic traits may be secondary to the observed somatotropic defect.

Mice with peripheral resistance to IGF-I or with low GH and IGF-I activity generally display altered glucose metabolism [3,10,33,34]. We therefore assessed glucose tolerance and insulin secretion in adult bIGF1RKO^+/−^ mice and found moderate, but significant hyperglycemic responses in males and females (Figure 5C). Moreover, although insulin secretory response 30 min after glucose injection appeared adequate in bIGF1RKO^+/−^ females, it was relatively insufficient in bIGF1RKO^+/−^ males (Figure 5D). The impaired control of glucose homeostasis and enlarged fat tissue in adult mutants indicate that the lack of IGF-I and GH progressively affected bIGF1RKO^+/−^ metabolism.

### bIGF1RKO^+/−^ Mice Live Longer Than Controls

**Figure 6:**
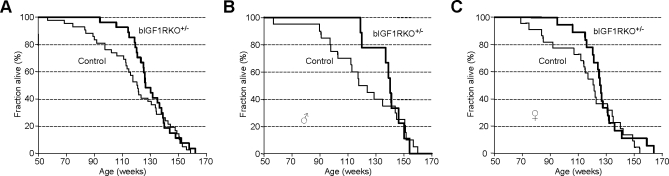
Lifespan Analysis (A) Survival curves show that bIGF1RKO ^+/−^ mice, on average, outlived their control littermates, although the maximum lifespan was similar for mutants and controls. (B and C) Separate survival curves for males and females. Mean lifespan for bIGF1RKO^+/−^ males was 966 ± 28 d versus 853 ± 43 d for male controls; Mean lifespan for bIGF1RKO^+/−^ females was 888 ± 27 d versus 821 ± 36 d for female controls. Differences were significant in Cox's test for males, females, and for both sexes combined when curves were censored before week 140, 130, and 135, respectively.

Constitutive inactivation of GHRH or GH receptors, as well as mutations impeding pituitary somatotroph development, increase longevity [6,9,35]. We therefore measured the lifespan of bIGF1RKO^+/−^ and control mice. Survival curves showed that bIGF1RKO^+/−^ mice had a significantly longer mean lifespan than control littermates (914 ± 21 d, *n* = 27 versus 836 ± 28 d, *n* = 42, *p* < 0.05) (Figure 6A). When we analyzed male and female mutants separately, we found similar increases in longevity (Figure 6B and 6C). However, the maximum life span was unchanged. The mortality rate for bIGF1RKO ^+/−^ mice up to 100 wk of age was six times lower than for controls (bIGF1RKO ^+/−^ 0.037 versus controls 0.238, *p* < 0.05). Thereafter, however, mortality rate of bIGF1RKO ^+/−^ increased sharply and was 55% higher for 121- to 140-wk-old bIGF1RKO ^+/−^ mice than controls in the same age range. Consequently, the inter-individual variation of lifespan was significantly lower for bIGF1RKO ^+/−^ mice than for controls (Figure S4). Thus, survival and mortality patterns in bIGF1RKO ^+/−^ mice under normal conditions were clearly affected. In contrast, we did not find differences in survival when mutants where challenged with acute oxidative stress (Figure S5), indicating that causes other than stress resistance were important for increased lifespan of bIGF1RKO ^+/−^ mice.

Post-mortem histopathology revealed a similarly diverse, heterogeneous panel of diseases in bIGF1RKO^+/−^ and control mice. To allow statistical evaluation despite relatively few observations, we categorized pathologies as inflammatory, degenerative, or tumor-related. The prevalence of inflammatory diseases was similar (bIGF1RKO^+/−^ 28% versus control mice 30%), but bIGF1RKO^+/−^ mice tended to develop fewer degenerative diseases (22% versus 27%, NS) and fewer tumors (44% versus 53%, NS). Notably, none of the bIGF1RKO^+/−^ animals over 130 wk of age showed pituitary tumors or hyperplasia, whereas 20% of the controls did, most probably a consequence of lifelong GHRH stimulation. However, as control animals had a fully normal lifespan, pituitary pathology was apparently not precipitating death. It was more likely that the reduced somatotropic tone in mutant mice protected them from death at an age by which a significant proportion of controls had already died. Concerning the prevalence of pathologies among those controls that die earlier than bIGF1RKO^+/−^, our data were nonconclusive, essentially due to the limited number of observations. Similarly, we could not find published information on early pathogenesis in B6/129 F1 hybrids. Finally, fewer bIGF1RKO^+/−^ individuals than controls presented multiple pathologies at death (17% versus 47%, *p* = 0.2), consistent with a lower disease burden in mutants.

## Discussion

### IGF and Somatotropic Plasticity

We showed that diminishing IGF-1R expression in the brain selectively reduced somatotropic function. This result suggests that brain IGF-1R controls the set-up of the GH/IGF axis, thereby modulating growth, energy metabolism, and lifespan. In contrast, complete absence of brain IGF-1R in the homozygous knockout produced a very different phenotype leading to increased IGF-I; thus, the phenotype is strongly dependent on IGF-1R gene dose. Any life-prolonging effects of early growth retardation in the homozygous mutant were subsequently suppressed, possibly by increased IGF-I. However, phenotypic alterations in the homozygote were so complex that comparing them with the heterozygote seemed a futile endeavor. Interestingly, brain-specific inactivation of IRS2, a major signal transduction molecule downstream from IGF-1R and insulin receptors, increased lifespan and altered nutrient homeostasis [12], while apparently not involving the somatotropic axis.

The heterozygous bIGF1RKO^+/−^ phenotype was somatotroph-specific and other neuroendocrine pathways were unaffected, implicating hypothalamic GHRH neurons. IGF receptors are indeed present in the arcuate nucleus where GHRH neurons reside. There is, however, little evidence for a specific developmental role for IGF-I in the regulation of GHRH neurons, and we cannot exclude the possibility that IGF-1R deletion from other brain areas affected GHRH neurons at a distance. Nevertheless, the similitude of bIGF1RKO^+/−^ mice with mutants lacking GHRH neurons [36] or GHRH receptors [9] also indicated that this phenotype could have been caused by reduced GHRH signals to the pituitary. Importantly, the number of GH-producing cells in bIGF1RKO^+/−^ pituitaries was only a fraction of that in controls. This finding was corroborated by immunohistochemistry (IHC) and concordant with the several-fold reduced pituitary GH content. GH depletion, in turn, explains the very low ALS concentration and absence of the pubertal surge in IGF-I. As the reduction in GHRH was proportionate to hypothalamic IGF-1R dose, GHRH action presumably triggers a disproportionate loss of GH cells. Several mechanisms may contribute to this: GHRH regulates the number of GH-positive cells, as well as GH production and secretion. Most likely, GHRH abundance must be above a certain threshold to stimulate hypothalamic-pituitary development and GH production. Moreover, not only GHRH but also SRIH neurons, which determine adult GH secretion, may be sensitive to IGF. SRIH was indeed above control concentrations in the adult bIGF1RKO^+/−^ hypothalamus, suggesting that coordinate GHRH and SRIH action on somatotrophs may have contributed to the observed over-proportionate effects.

GHRH and SRIH accumulate in nerve endings at the ME, and are liberated into portal veins to regulate pituitary GH. During development, somatotroph proliferation and differentiation depend on local GHRH. In bIGF1RKO^+/−^ hypothalamus, immunoreactive GHRH but not SRIH was less abundant than in controls. This difference in amount suggests a shift from GHRH to SRIH stimulation at the ME and has possible functional implications for pituitary development, e.g. via Pit-1. Early lack of GHRH in ME, as observed here, may be due to less GHRH neurons, fewer nerve endings, or diminished GHRH. Although we cannot conclude from our experiments, IGF receptors in the ME of adult bIGF1RKO^+/−^ appeared to be less phosphorylated than those in the rest of the hypothalamus (38.8 ± 2.6 versus 59.0 ± 4.9, arbitrary units, *p* = 0.005, *n* = 8; no difference in control mice; Figure S3B). Thus, ME may participate in somatotropic control, for instance by sensing circulating IGF-I. The ME also harbors numerous tanycytes, glia specialized in IGF-I transport to the brain, that may contribute to the modulation of somatotropic signals by acting on the maturation of GHRH neurons. Our various findings combine to indicate that IGF-I, the principal peripheral mediator of GH action, itself plays a major role in the development of the GH/IGF axis.

Cells in small organisms communicate trophic status via paracrine insulin-like signals [37]. Our results suggest that IGF-I may have conserved a role of this type in complex organisms and act, in particular, during development as a trophic signal that represents numerous, distant tissues [38]. The neuroendocrine network of GHRH, SRIH, and GH cells evolved in vertebrates as a central regulator that coordinates growth of heterogeneous cell populations. Here, we describe a mechanism that determines the individual postnatal trajectory of the somatotropic axis and its adult set point. Somatotropic plasticity, as we observed here, may have evolved to adjust growth to environmental resources. We further demonstrated that early dietary restriction can trigger a neuroendocrine response similar to brain-specific IGF-1R knockout (see Text S1, Supplementary Results and Figure S6). Both genetic and nutritional intervention led to growth deficits and a lifelong reduction in endocrine IGF-I. Thus, it appears that a fairly direct hormonal path connects nutrition, somatotropic hormones and growth through a positive feedback (Figure S7). This view is supported by recent evidence that AT, a major indicator of postnatal nutrition, is an important source of IGF-I [39]. Also, the phylogenetically related insulin receptor (IR) on pancreatic β-cells controls development of the insulin/IR hormone axis in a very similar manner [3,4].

Heterozygous inactivation of brain IGF-1R led to a consistent somatotropic deficit, but without any detectable effects on other brain functions. Interestingly, we found that in the bIGF1RKO^+/−^ brain, the remaining IGF receptors were overphosphorylated in the cerebral cortex (120% ± 5% of controls, *n* = 16, *p* < 0.05; Figure S3A) but not in hypothalamus. This cortical IGF-1R activation may be due to a compensatory local increase of ligand. In contrast, complete absence of IGF-1R in bIGF1RKO^−/−^ mutants (Figure S1) resulted in microcephaly, indicative of a neurotrophic role for IGF-1R. We conclude that the IGF-1R gene is heterosufficient with respect to neurotrophic action, but heteroinsufficient with regard to its role in regulating somatotropic plasticity.

We showed that downregulation of somatotropic hormones postponed mortality and increased mean lifespan. The underlying developmental mechanism suggested that neuroendocrine control of lifespan may be physiologically relevant in mammals. Indeed, somatotropic plasticity may confer an evolutionary advantage: adaptation of individual body size to available resources would improve fitness in the face of environmental changes; control of size, energy metabolism, and lifespan contribute to preserving vital resources and to maintaining genetic diversity during periods of shortage. Therefore, the evolutionary significance of somatotropic plasticity may be that it cushions otherwise deleterious effects of selective pressure.

The Barker hypothesis and predictive adaptive response (PAR) hypothesis claim that changes in development can have late consequences for health, modify mortality and lifespan, and entail a risk of maladaptation later in life, if environmental conditions differ grossly from those that led to the initial changes [40,41]. This may apply to bIGF1RKO^+/−^ mice, adapted to a poor environment (little IGF signaling to the brain), when food was available ad libitum. The resulting dyslipidemia and hyperglycemia in bIGF1RKO^+/−^ mutants may explain the rapid increase in late-life mortality and unusual combination of extended mean but normal maximum lifespan. Similar patterns of survival have been described in other aging models: in Caenorhabditis elegans, ablation of ASI sensory neurons that modulate DAF-2 pathways in response to environment, increased mean, but not maximum lifespan [27]; in *Drosophila*, targeted expression of human uncoupling protein 2 in adult neurons [42], or inhibition of p53 specifically in the nervous system [43], extended mean, but not maximum, lifespan. Common to these models is that the mutations were targeted to the nervous system, and it is possible that the perturbed neuronal responsiveness to environmental cues is the primary cause of prolonged lifespan in these models.

An alternative interpretation of our findings with bIGF1RKO^+/−^ mice is possible. The average lifespan of the mutants was longer than controls, but its variability was half than that for controls. Thus, individuals on the short-lived edge of the normal lifespan distribution (see Figures 6 and S4) benefit most from reduced growth hormone and IGF-I: they respond with a lifespan extension of nearly 40%. This finding raises the question of why genetically homogeneous wild-type populations display large variability in lifespan. A current explanation is that initially small stochastic differences between individuals are subsequently amplified during development and adult life; however, the mechanism for this remains unclear. Our data indicate that a full complement of brain IGF-1R is required for generating a high somatotropic tone, and this eventually increases the risk of dying early. This mechanism may produce much of the lifespan heterogeneity observed in wild-type mice. Remarkably, the variability of plasma GH was several-fold higher in controls than in bIGF1RKO^+/−^ mice, the mutants having continuously low GH levels (M. Holzenberger, L. Kappeler, and A. Bekaert, unpublished data). There are indeed other mammalian species where short lifespan can be related to elevated somatotropic signaling, and vice-versa. In dogs, small size and low IGF-I are determined by one particular IGF-I allele [44], and small breeds live twice as long as large breeds [45]. Van Heemst et al. [19] investigated the relationship between somatotropic function, adult height, and longevity in humans, and found that in women, genetically determined low GH and IGF-I signaling was beneficial for old age survival. Recently, functionally relevant IGF-1R mutations were isolated from human centenarians [17]. Persisting high GH in acromegalic patients after surgical treatment is predictive of premature death [46], and elevated IGF-I in elderly individuals correlates with increased mortality [47]. Furthermore, the average lifespan of people with pituitary gigantism (mean height 236 cm, *n* = 28) is only 44 y, roughly 20 y below the corresponding historical adult life expectancy. It would be extremely valuable to measure the consequences of somatotropic tone for human health and longevity in a variety of contexts to clarify these important issues.

## Methods

### Mouse genetics.

*Igf1r^flox^* mice [48,49] were maintained in a 129/Sv (129) genetic background and also backcrossed to C57BL/6 (B6) for >15 generations. The Nestin-Cre (*NesCre*) transgene (maintained in B6 for >15 generations) produces Cre recombinase in neural and glial precursors during early neural development [50]. By mating *Igf1r^flox/+^* females (B6) with *NesCre^+/0^* males we generated *Igf1r^flox/+^;NesCre^+/0^* double mutants in a B6 background. These were mated with *Igf1r^flox/flox^* females (129) to produce experimental cohorts as fully reproducible F1 generations of B6/129 hybrid genetic background, composed of hetero- (+/−) and homozygous (−/−) brain-specific IGF-1R knockout mice (bIGF1RKO) and their littermate controls. F1 hybrids from pure inbred strains combine two advantages: reproducibility of genetic composition and so-called hybrid vigor, i.e. the absence of phenotypic defects that affect pure inbred strains. F1 hybrids are generally long-lived, like mice from mixed genetic backgrounds and some pure inbred strains, including C57BL/6. We conducted experiments according to institutional guidelines for the care of laboratory animals.

### Mice.

Animals lived under SPF conditions in individually ventilated cages at 23 °C, with a 14/10-h light/dark cycle and free access to water and a commercial rodent diet (49% carbohydrate, 24% protein, 5% lipid, 12% humidity, 10% mineral, and fiber). Mice were separated from mothers on day 30 and housed six males or six females per cage, with both control and mutant genotypes in each cage. Cages were equipped with a mouse house to enhance social interaction and prevent male aggressiveness. We produced four cohorts. In cohort 1 (105 bIGF1RKO^+/−^, 116 control males) we analyzed somatotropic development. To minimize litter effects, they were trimmed to four to six pups per mother. For experiments, mutants and controls were always litter-matched and each group was composed of animals from at least three different litters. In cohort 2 (57 bIGF1RKO^+/−^, 51 controls) we studied growth, metabolism, behavior, hormones, and body composition. In cohort 3 (45 bIGF1RKO^+/−^, 60 controls) we analyzed additional blood variables, female fertility, and ovarian function. Mice of cohort 4 (27 bIGF1RKO^+/−^, 19 bIGF1RKO^−/−^, 42 controls) were checked daily, but otherwise left undisturbed until they died naturally. Single surviving females were housed with neighbors. Necropsy was performed on all animals. Brain, heart, lung, liver, kidney, and spleen were dissected post mortem and fixed in formalin for histology. We determined major pathologies present at death for 62 of the 88 animals.

### Postnatal growth.

Mice were weighed daily until the age of 6 wk and weekly thereafter. For growth curves, we used sliding means of current weight and weight on the preceding and subsequent day (or week, for age >6 wk).

### Fertility and reproduction.

Estrous cycle length was determined by daily monitoring of vaginal smear histology for 3 to 4 wk. Blood and ovaries were sampled between 11 a.m. and 14 p.m. on the pro-estrus day under pentobarbital anesthesia. Mean litter size was calculated from matings between bIGF1RKO^+/−^ females and wild-type males.

### Body temperature.

Rectal temperature was determined on three consecutive days. Measurement began within 10 s of immobilization, to avoid artifacts of stress-induced thermogenesis.

### Histology and immunohistochemistry.

LacZ staining was performed on parasagittal 14-μm-thick cryosections from 2-wk-old double transgenic *NesCre^+/0^;Rosa26R^+/0^* mice [33]. *Rosa26R^+/0^* littermates served as negative controls. Sections were fixed in PAF, stained with X-Gal overnight and counterstained with orange G. Inguinal fat pads from males were quickly frozen and cryosections (50 μm; −30 °C) fixed in 4% paraformaldehyde (PAF) for hematoxylin staining. Digital micrographs in visible light were taken with an Olympus BX51 microscope. We detected GH in the pituitary, and GHRH and SRIH in the ME using standard fluorescent IHC. Sections 18 μm thick were fixed in PAF and incubated overnight with rabbit anti-GH (NIH-NIDDK), rabbit anti-GHRH, or goat anti-SRIH antibodies (Santa Cruz). Secondary antibodies were Alexa 546 goat anti-rabbit and Alexa 488 donkey anti-goat (Molecular Probes). For fluorescent IHC we used an Olympus BX612 microscope. To compare the relative intensities of IHC signals for GH, GHRH, and SRIH between mutants and controls, we processed all samples under identical conditions with respect to anatomical location, antibody incubation, laser intensity, and CCD image acquisition, in particular using identical parameters of signal integration. For each animal, data obtained from ten different tissue sections were averaged. Means were then compared between groups using Student's *t*-test.

### Western blotting.

Immunoprecipitation and western blotting were as described previously [51]. Antibodies used were anti-IGF-1R β-subunit (C20, Santa Cruz), anti-phospho-tyrosine (PY20, Transduction Laboratories), anti-mouse GHR (from G. Gudmundur and F. Talamantes), anti-ALS (AF1436, R&D Systems), anti-mouse P-Jak2 Tyr1007/1008 (Cell Signalling/Ozyme), anti-β-tubulin (Oncogene Research), and anti-β-actin (Sigma). We confirmed equal loading for each immunoblot. Bound antibody was revealed using peroxidase-conjugated secondary antibodies and ECL (Amersham Pharmacia Biotech). Signals were quantified using MacBas 2.5 (Fuji) or film autoradiography and NIH Image (for ALS).

### Biochemistry.

From blood samples we determined total bilirubin, HDL, and total cholesterol, triglycerides, creatinine, glucose, lactate, total protein, urea and total antioxidant status, using an Olympus Diagnostic Automat. We tested glucose tolerance in animals fasted for 14 h by intraperitoneal injection of 20%-D-glucose (2 g/kg body weight): glucose was assayed in tail blood at 0, 15, 30, 60, and 120 min using a hand-held photometer (Lifescan Glucotouch); insulinemia was measured at 0 and 30 min. We used RIA to determine plasma IGF-I (Diagnostic Systems Laboratories), insulin, leptin (both from Linco), and T4 (MP Biomedical). Estradiol concentrations were determined by RIA in 150 μl of mouse pro-estrus serum (Diasorin). Pituitary GH content was measured using a rat-specific RIA (Linco). GHRH and SRIH were quantified in sonicated hypothalami by RIA [52]. The BCAssay (Uptima UP40840A, Interchim) was used to determine pituitary and hypothalamic protein content.

### GH receptor stimulation.

Human recombinant GH (Serono) was injected into the portal vein of mice (0.5 μg/g body weight) under isoflurane anesthesia (Abbott Laboratories). Liver biopsies, taken at the time of injection and 2, 5, and 20 min later were immediately frozen for protein extraction.

### Real-Time Reverse Transcription-PCR.

Total RNA from hypothalami and pituitaries were extracted with phenol-chloroform (RNAble; Eurobio) and aliquots of 1 μg were reverse transcribed (Transcriptor; Roche) using random hexamers (Promega) in the presence of RNAsine (Promega). Duplicates of 10 ng cDNA were used for real-time PCR amplification in an Applied Biosystem's PCR System 7300 and its reagents (GHRH, Mm00439100_m1; SRIH, Mm00436671_m1; NPY, Mm00445771_m1; Pit-1, Mm00476852_m1; GH, Mm00433590_g1; PRL, Mm005599949_m1; TSH-β, Mm00437190_m1; LH-β, Mm00656868_q1; β-actin, 4352933E; 18S rRNA, 4333760F). We quantified mRNA using standard curves generated with a control sample and values were normalized to values for housekeeping gene mRNAs.

### Statistics.

For group comparison, we used two-tailed Student's *t*-test. Means are expressed ± SEM. We determined the significance of survival curves by Cox's test, and used paired *t*, Mann-Whitney, and Chi^2^ tests where indicated. Levels of significance were *, *p* < 0.05; **, *p* < 0.01; ***, *p* < 0.001; NS, not significant (*p* ≥ 0.05).

## Supporting Information

Figure S1 Phenotype of Mice with Homozygous IGF-1R Knockout in the CNS (bIGF1RKO^−/−^) Homozygous knockout brains contained 11% of control IGF-1R levels (29 ± 2 fmol/mg versus 278 ± 22 fmol/mg, *n* = 14, determined by in vitro ligand-binding assay; unpublished data) stemming from cells of non-neuroepithelial origin, including blood vessels and meninges.
(A) Unlike heterozygous bIGF1RKO^+/−^, the homozygotes were growth retarded at birth (∼80% of normal birth weight), and their cranium was flat.
(B) bIGF1RKO^−/−^ mice were viable, but grew slowly (here at 6 wk of age).
(C) Frontal brain sections from adult bIGF1RKO^−/−^ mutants revealed marked microcephaly.
(D) After severe growth retardation, bIGF1RKO^−/−^ mice caught up with normal size at around 4 mo (left panel) and body weight at 12 mo was not different from control littermates (right panel; data represent males).
(E) bIGF1RKO^−/−^ mice showed the same average lifespan as controls (835 d ± 34 d, *n* = 19 control 836 d ± 28 d, *n* = 42). Male and female data were very similar and thus combined.
(F) Adult bIGF1RKO^−/−^ mice had elevated fasting glycemia and were markedly glucose intolerant.
(G) Serum IGF-I was significantly decreased at 4 wk, but increased at 8 wk. IGF-I levels continued to be 30%–40% increased throughout adult life (unpublished data). bIGF1RKO^−/−^ did not perform in standard behavioral testing.
(308 KB PDF)

Figure S2 Activity, Nutrition, and Behavioral Analysis (A) Circadian profiles of physical activity were identical in bIGF1RKO^+/−^ and control mice. Males and females behaved similarly and are shown together.
(B) Daily food (left) and water consumption (right) did not differ between bIGF1RKO^+/−^ mice and controls.
(C) Short term spatial memory was unaffected in bIGF1RKO^+/−^ males (right) and females (left): in all groups, mice preferred to explore the new area in a Y-maze.
(D) bIGF1RKO^+/−^ mice behaved similarly as controls in the open field test (unpublished data). However, exploratory behavior was selectively impaired in bIGF1RKO^+/−^ males as shown by the novel object test.
(E) When testing anxiety in an O-maze, bIGF1RKO^+/−^ females and controls behaved similarly, whereas bIGF1RKO^+/−^ males were less anxious (*p* < 0.05, Mann-Whitney test). Note that males of B6/129-F1 hybrid genetic background generally display higher levels of anxiety than females [2]. Collectively, we did not find significant behavioral differences, other than slightly impaired exploration and reduced anxiety in bIGF1RKO^+/−^ males.
(92 KB PDF)

Figure S3 Activation of IGF-1R in Adult bIGF1RKO^+/−^ Brain (A) IGF-1R was significantly underphoshorylated in mutant muscle (hind-limb) and hypothalamus, and overphosphorylated in cerebral cortex.
(B) When ME was separated from the rest of the hypothalamus, underphophorylation located to ME only. * *p* < 0.05, ** *p* < 0.01, *** *p* < 0.001; (au), arbitrary units.
(52 KB PDF)

Figure S4 Variation of Lifespan and Mortality in bIGF1RKO^+/−^ (Blue) and Control Mice (Red) (A) Proportion of mice that died within a given age range. The distribution was significantly narrower in bIGF1RKO^+/−^ mice compared to controls (*p* < 0.01; *F*-test). 80% of the mutant mice died within a 33-wk interval, whereas the same proportion of control deaths occurred over a 69-wk period.
(B) Mortality in control mice showed a normal increase with age. Mortality in bIGF1RKO^+/−^ mutants occurred much later but increased rapidly after 120 wk. Mortality above 150 wk was 1.0 for both groups.
(52 KB PDF)

Figure S5 Click here for additional data file. Short-Term Survival of 3-Mo-old bIGF1RKO+/− Mice Challenged with Oxidative Stress bIGF1RKO^+/−^ and Control Males Received 60 mg/kg diquat (A) (*n* = 13 and 10) or 500 mg/kg Acetaminophen (B) (*n* = 13 and 14) as a Single Intraperitoneal Injection
No statistically significant differences existed between groups. Methods are as described in [10]; if more than one animal died within the same hour they were represented together in one data point.
(43 KB PDF)

Figure S6 Early Postnatal Nutrient Restriction in Wild Type Mice Inhibited the Development of Somatotropic Function (A) Nutrient restriction was achieved by increasing the litter size at birth to ten sucklings per mother, resulting in less mother's milk, and comparing with litters trimmed to six sucklings, which ensured normal nutrient supply. After 2 wk, all mice were fed with rodent chow ad libitum. Growth of restricted mice was progressively delayed (*p* < 0.001, from day 2 onwards).
(B) Left: early nutrient restriction reversibly diminished leptinemia. Glycemia and other nutritional markers behaved similarly (unpublished data). Right: hypothalamic GHRH expression (relative to β-actin) was significantly decreased in restricted mice at 10 d.
(C) Pituitary GH content was conspicuously low in restricted mice at 20 d, while plasma IGF-I was decreased under nutrient restriction and also thereafter, under ad libitum feeding.
(76 KB PDF)

Figure S7 Functional Development of the Somatotropic Axis Depends on IGF-1R Signaling Collectively, our results suggest that the functional development of the somatotropic axis depends on IGF-1R signaling (left drawing). Reduced IGF-I signaling in the brain during early life, as with bIGF1RKO^+/−^ mice, retards somatotropic development and leads to adult GH deficiency. Reversely, it is possible that IGF-I, produced as a normal response to nutrition, stimulates brain IGF-1R and induces GHRH and Pit-1 production, eventually translating as a positive feedback into increased GH and IGF-I [3]. This would be different from adult physiology (right drawing), characterized by negative feedback of peripheral IGF-I to hypothalamic IGF-1R, thereby inhibiting GHRH and GH secretion [34,4,5]. It seems possible that this neuroendocrine plasticity of somatotropic function during early postnatal development determines individual endocrine life trajectories.
(45 KB PDF)

Table S1 Adult Body Composition at 10 mo of Age (56 KB DOC)

Table S2 AT in Percent of Body Weight (52 KB DOC)

Table S3 Blood Biochemistry at 4 mo of Age (47 KB DOC)

Text S1 Supplementary Results (74 KB DOC)

## Abbreviations

ALS: acid labile subunit
AT: adipose tissue
bIGF1RKO: brain-specific IGF-1 receptor knockout
CNS: central nervous system
GH: growth hormone
GHRH: growth hormone releasing hormone
IGF: insulin-like growth factor
IGF-1R: insulin-like growth factor receptor
IHC: immunohistochemistry
IRS: insulin-receptor substrate
ME: median eminence
NS: not significant
SRIH: somatostatin
