# Supplementary figures and images for: Brain IGF-1 Receptors Control Mammalian Growth and Lifespan through a Neuroendocrine Mechanism
Source: PLoS Biol. 2008 Oct 28;6(10):e254. doi: 10.1371/journal.pbio.0060254 (PMC2573928; doi:10.1371/journal.pbio.0060254)

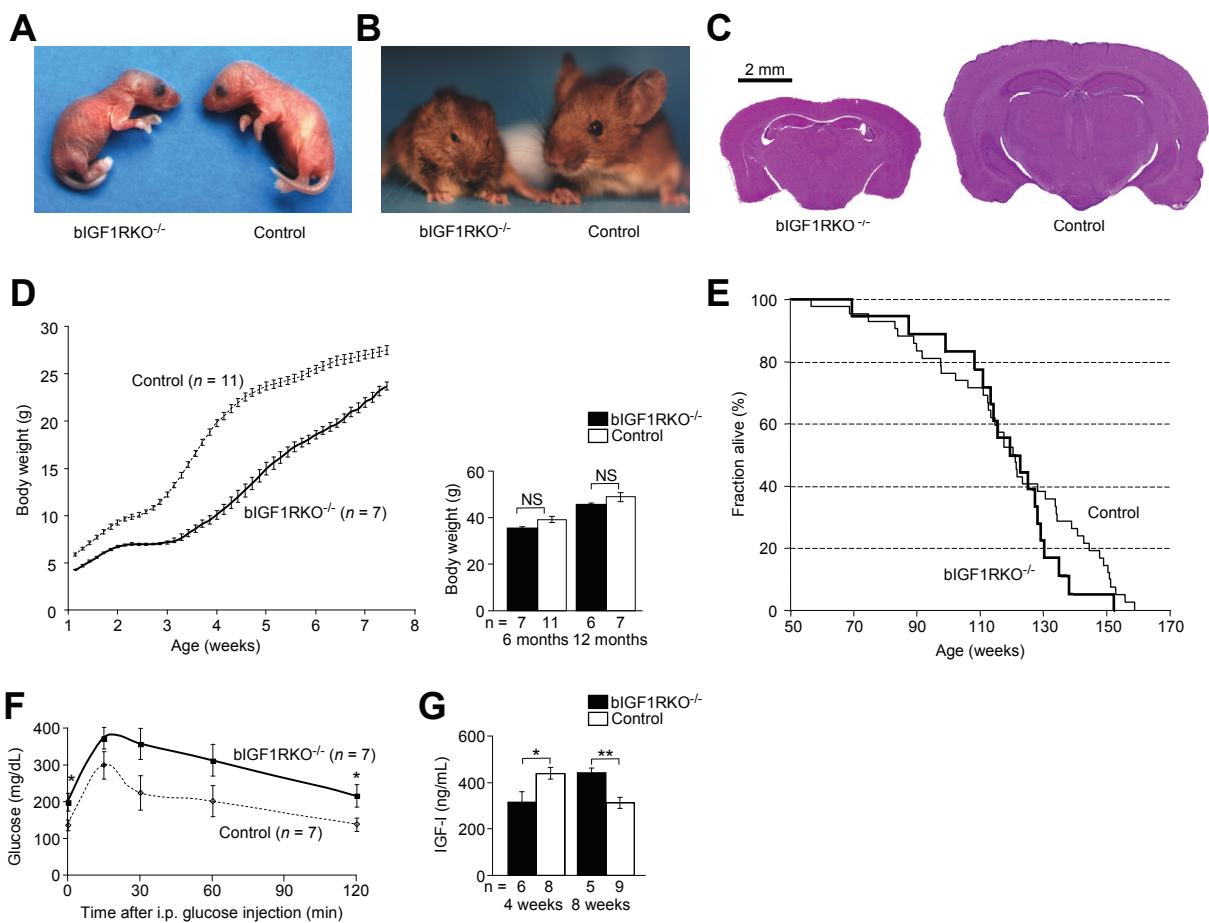

Supplement: Figure S1 — Homozygous knockout brains contained 11% of control IGF-1R levels (29 ± 2 fmol/mg versus 278 ± 22 fmol/mg, n = 14, determined by in vitro ligand-binding assay; unpublished data) stemming from cells of non-neuroepithelial origin, including blood vessels and meninges. (A) Unlike heterozygous bIGF1RKO+/−, the homozygotes were growth retarded at birth (∼80% of normal birth weight), and their cranium was flat. (B) bIGF1RKO−/− mice were viable, but grew slowly (here at 6 wk of age). (C) Frontal brain sections from adult bIGF1RKO−/− mutants revealed marked microcephaly. (D) After severe growth retardation, bIGF1RKO−/− mice caught up with normal size at around 4 mo (left panel) and body weight at 12 mo was not different from control littermates (right panel; data represent males). (E) bIGF1RKO−/− mice showed the same average lifespan as controls (835 d ± 34 d, n = 19 control 836 d ± 28 d, n = 42). Male and female data were very similar and thus combined. (F) Adult bIGF1RKO−/− mice had elevated fasting glycemia and were markedly glucose intolerant. (G) Serum IGF-I was significantly decreased at 4 wk, but increased at 8 wk. IGF-I levels continued to be 30%–40% increased throughout adult life (unpublished data). bIGF1RKO−/− did not perform in standard behavioral testing. (308 KB PDF) [file pbio.0060254.sg001.pdf]

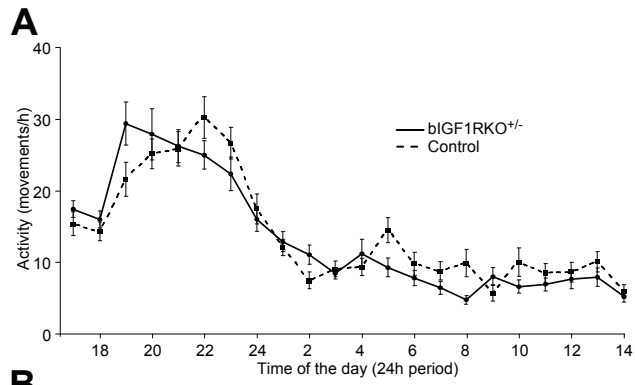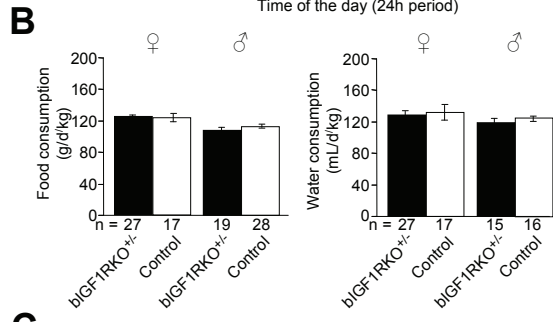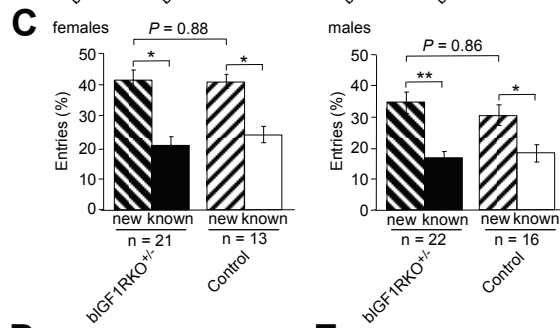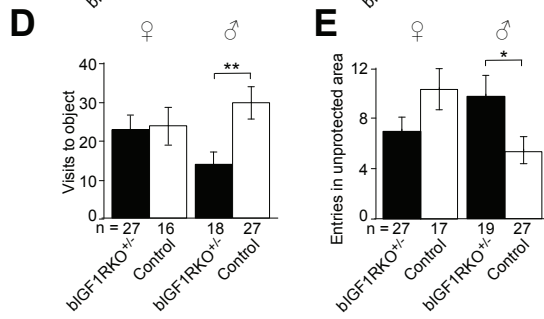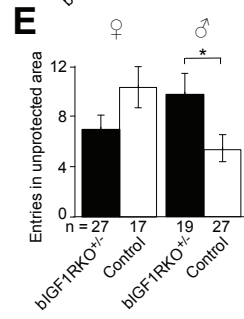

Supplement: Figure S2 — (A) Circadian profiles of physical activity were identical in bIGF1RKO+/− and control mice. Males and females behaved similarly and are shown together. (B) Daily food (left) and water consumption (right) did not differ between bIGF1RKO+/− mice and controls. (C) Short term spatial memory was unaffected in bIGF1RKO+/− males (right) and females (left): in all groups, mice preferred to explore the new area in a Y-maze. (D) bIGF1RKO+/− mice behaved similarly as controls in the open field test (unpublished data). However, exploratory behavior was selectively impaired in bIGF1RKO+/− males as shown by the novel object test. (E) When testing anxiety in an O-maze, bIGF1RKO+/− females and controls behaved similarly, whereas bIGF1RKO+/− males were less anxious (p < 0.05, Mann-Whitney test). Note that males of B6/129-F1 hybrid genetic background generally display higher levels of anxiety than females [2]. Collectively, we did not find significant behavioral differences, other than slightly impaired exploration and reduced anxiety in bIGF1RKO+/− males. (92 KB PDF) [file pbio.0060254.sg002.pdf]

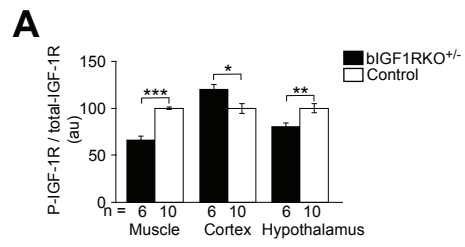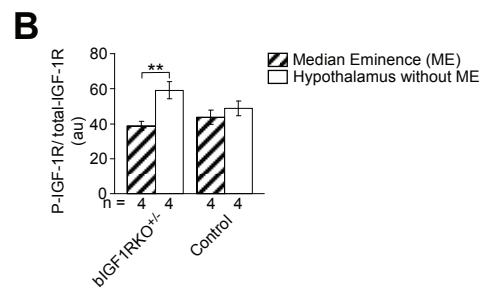

Supplement: Figure S3 — (A) IGF-1R was significantly underphoshorylated in mutant muscle (hind-limb) and hypothalamus, and overphosphorylated in cerebral cortex. (B) When ME was separated from the rest of the hypothalamus, underphophorylation located to ME only. * p < 0.05, ** p < 0.01, *** p < 0.001; (au), arbitrary units. (52 KB PDF) [file pbio.0060254.sg003.pdf]

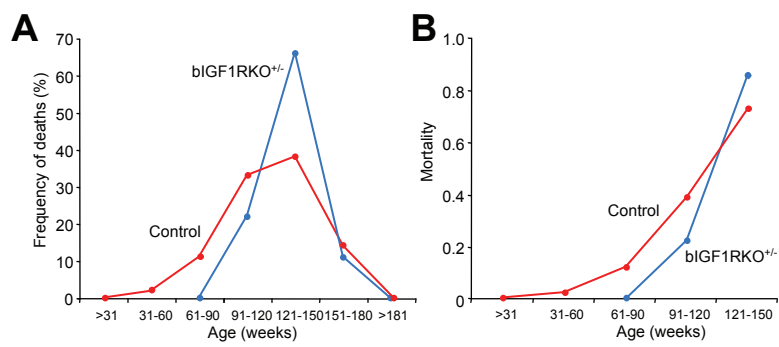

Supplement: Figure S4 — (A) Proportion of mice that died within a given age range. The distribution was significantly narrower in bIGF1RKO+/− mice compared to controls (p < 0.01; F-test). 80% of the mutant mice died within a 33-wk interval, whereas the same proportion of control deaths occurred over a 69-wk period. (B) Mortality in control mice showed a normal increase with age. Mortality in bIGF1RKO+/− mutants occurred much later but increased rapidly after 120 wk. Mortality above 150 wk was 1.0 for both groups. (52 KB PDF) [file pbio.0060254.sg004.pdf]

**A**

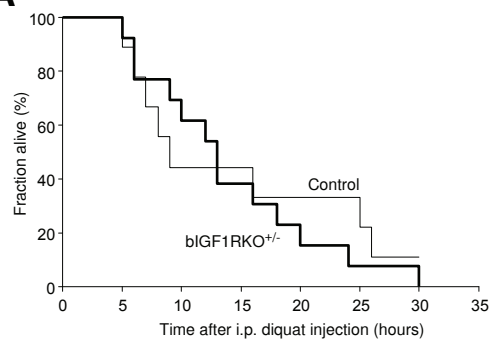

**B**

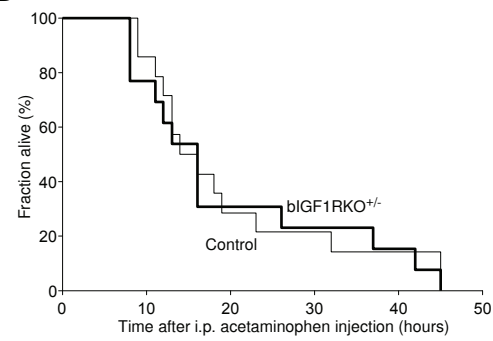

Supplement: Figure S5 [file pbio.0060254.sg005.pdf]

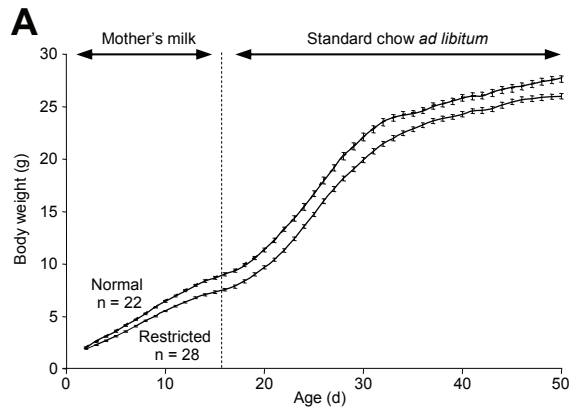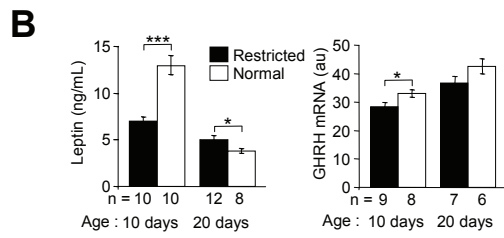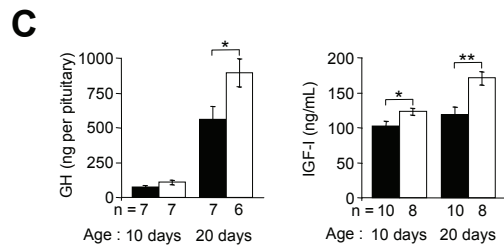

Supplement: Figure S6 — (A) Nutrient restriction was achieved by increasing the litter size at birth to ten sucklings per mother, resulting in less mother's milk, and comparing with litters trimmed to six sucklings, which ensured normal nutrient supply. After 2 wk, all mice were fed with rodent chow ad libitum. Growth of restricted mice was progressively delayed (p < 0.001, from day 2 onwards). (B) Left: early nutrient restriction reversibly diminished leptinemia. Glycemia and other nutritional markers behaved similarly (unpublished data). Right: hypothalamic GHRH expression (relative to β-actin) was significantly decreased in restricted mice at 10 d. (C) Pituitary GH content was conspicuously low in restricted mice at 20 d, while plasma IGF-I was decreased under nutrient restriction and also thereafter, under ad libitum feeding. (76 KB PDF) [file pbio.0060254.sg006.pdf]

**Early postnatal**

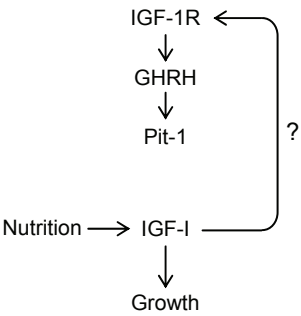

**Adult**

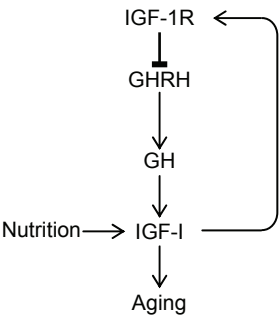

Supplement: Figure S7 — Collectively, our results suggest that the functional development of the somatotropic axis depends on IGF-1R signaling (left drawing). Reduced IGF-I signaling in the brain during early life, as with bIGF1RKO+/− mice, retards somatotropic development and leads to adult GH deficiency. Reversely, it is possible that IGF-I, produced as a normal response to nutrition, stimulates brain IGF-1R and induces GHRH and Pit-1 production, eventually translating as a positive feedback into increased GH and IGF-I [3]. This would be different from adult physiology (right drawing), characterized by negative feedback of peripheral IGF-I to hypothalamic IGF-1R, thereby inhibiting GHRH and GH secretion [34,4,5]. It seems possible that this neuroendocrine plasticity of somatotropic function during early postnatal development determines individual endocrine life trajectories. (45 KB PDF) [file pbio.0060254.sg007.pdf]
