# Supplementary material for: Brain IGF-1 Receptors Control Mammalian Growth and Lifespan through a Neuroendocrine Mechanism
Source: PLoS Biol. 2008 Oct 28;6(10):e254. doi: 10.1371/journal.pbio.0060254 (PMC2573928; doi:10.1371/journal.pbio.0060254)
Supplement: Table S1 — (56 KB DOC) [file pbio.0060254.st001.doc]

| **Supplementary Table 1 Adult body composition at 10 months of age** | | | | | | | | | | | | | | | |
| --- | --- | --- | --- | --- | --- | --- | --- | --- | --- | --- | --- | --- | --- | --- | --- |
|  | Males | | | | | |  | | Females | | | | | | |
|  | bIGF1RKO^+/- 1^ | | Control ^2^ | | B/C | |  | | bIGF1RKO^+/- 3^ | | | | Control ^4^ | | B/C |
| Body weight (g) | 38.6 | ± 1.2 * | 41.9 | ± 0.6 | | *92%* | |  | | 31.8 | ± 0.5 | 31.3 | | ± 0.6 | *102%* |
| Body length (cm) | 11.0 | ± 0.1 ** | 11.7 | ± 0.1 | | *94%* | |  | | 10.9 | ± 0.1 ** | 11.3 | | ± 0.1 | *96%* |
| Brain (mg) | 472 | ± 3 ** | 528 | ± 3 | | *89%* | |  | | 486 | ± 5 ** | 540 | | ± 7 | *90%* |
| Pituitary gland (mg) | 1.50 | ± 0.07 ** | 2.52 | ± 0.26 | | *60%* | |  | | 2.05 | ± 0.14 ** | 3.04 | | ± 0.11 | *67%* |
| Heart (mg) | 148 | ± 4 ** | 211 | ± 5 | | *70%* | |  | | 117 | ± 2 *** | 141 | | ± 3 | *83%* |
| Lungs (mg) | 188 | ± 8 * | 217 | ± 5 | | *87%* | |  | | 179 | ± 7 | 185 | | ± 7 | *96%* |
| Liver (g) | 1.39 | ± 0.05 ** | 1.79 | ± 0.03 | | *77%* | |  | | 0.98 | ± 0.02 * | 1.09 | | ± 0.04 | *90%* |
| Spleen (mg) | 99 | ± 10 *^P^*^=0.05^ | 121 | ± 6 | | *82%* | |  | | 108 | ± 4 | 112 | | ± 6 | *97%* |
| Kidney (mg) | 390 | ± 9 ** | 570 | ± 7 | | *68%* | |  | | 325 | ± 6 ** | 386 | | ± 9 | *84%* |
| Muscle (g) | 5.39 | ± 0.12 ** | 6.76 | ± 0.15 | | *80%* | |  | | 4.83 | ± 0.09 * | 5.13 | | ± 0.11 | *94%* |
| Gonads (mg)^5^ | 208 | ± 4 *** | 226 | ± 4 | | *92%* | |  | | 11.8 | ± 0.9 | 13.4 | | ± 0.9 | *88%* |
| Carcass (g) | 11.3 | ± 0.2 ** | 13.7 | ± 0.2 | | *83%* | |  | | 9.0 | ± 0.2 * | 10.0 | | ± 0.2 | *90%* |

^1^: *n* = 19; ^2^: *n* = 28; ^3^: *n* = 25; ^4^: *n* = 17.

^5^: Measured at 7 months of age

**P* < 0.05; ***P* < 0.01; *** *P* < 0.001; using Student’s *t*-test.
