# Supplementary material for: Brain IGF-1 Receptors Control Mammalian Growth and Lifespan through a Neuroendocrine Mechanism
Source: PLoS Biol. 2008 Oct 28;6(10):e254. doi: 10.1371/journal.pbio.0060254 (PMC2573928; doi:10.1371/journal.pbio.0060254)
Supplement: Table S2 — (52 KB DOC) [file pbio.0060254.st002.doc]

| **Supplementary Table 2 Adipose tissue (AT) in percent of body weight** | | | | | | | | | | | |
| --- | --- | --- | --- | --- | --- | --- | --- | --- | --- | --- | --- |
|  | Males | | | | Females | | | | | | |
|  | bIGF1RKO^+/- 1^ | | Control ^2^ | | bIGF1RKO^+/- 3^ | | | Control ^4^ | | | |
| Subcutaneous AT^5^ | 10.63 | ± 0.54 *** | 7.40 | ± 0.44 | 8.55 | | ± 0.50 ** | | 5.74 | | ± 0.62 |
| - inguinal/dorsolumbar | 6.49 | ± 0.39 *** | 4.50 | ± 0.29 | 4.85 | | ± 0.27 ** | | 3.36 | | ± 0.36 |
| - interscapular | 4.14 | ± 0.42 * | 2.91 | ± 0.24 | 3.70 | | ± 0.29 ** | | 2.39 | | ± 0.34 |
| Visceral AT | 7.78 | ± 0.16 * | 8.69 | ± 0.25 | 8.13 | | ± 0.24 ** | | 5.96 | | ± 0.53 |
| - gonadal | 4.34 | ± 0.11 * | 4.79 | ± 0.15 | 4.52 | | ± 0.16 *** | | 3.24 | | ± 0.27 |
| - mesenteric | 1.84 | ± 0.10 ** | 2.40 | ± 0.13 | 1.84 | | ± 0.09 | | 1.55 | | ± 0.17 |
| - perirenal | 1.60 | ± 0.05 | 1.51 | ± 0.07 | 1.78 | | ± 0.09 ** | | 1.17 | | ± 0.16 |
| Total AT (subcut + visc) | 18.41 | ± 0.58 * | 16.09 | ± 0.61 | 16.69 | ± 0.70 *** | | 11.70 | | ± 1.13 | |

^1^: *n* = 9; ^2^: *n* = 12; ^3^: *n* = 13; ^4^: *n* = 11.

^5^: Subcutaneous AT is the sum of inguinal/dorsolumbar and interscapular fat pads.

^6^: Visceral AT is the sum of gonadal, mesenteric and perirenal fat pads.

**P* < 0.05; ** *P* < 0.01; ****P* < 0.001; using Student’s *t*-test.
