# Supplementary material for: Brain IGF-1 Receptors Control Mammalian Growth and Lifespan through a Neuroendocrine Mechanism
Source: PLoS Biol. 2008 Oct 28;6(10):e254. doi: 10.1371/journal.pbio.0060254 (PMC2573928; doi:10.1371/journal.pbio.0060254)
Supplement: Table S3 — (47 KB DOC) [file pbio.0060254.st003.doc]

| **Supplementary Table 3 Blood biochemistry at 4 months of age** | | | | | | | | | | |
| --- | --- | --- | --- | --- | --- | --- | --- | --- | --- | --- |
|  | Males | | | |  | | Females | | | |
|  | bIGF1RKO^+/- 1^ | | Control ^2^ | |  | | bIGF1RKO^+/- 3^ | | Control ^4^ | |
| Triglycerides (mmol/L) | 1.26 | ± 0.09 | 1.28 | ± 0.08 |  | 0.93 | | ± 0.03 | 1.02 | ± 0.05 |
| Cholesterol (mmol/L) | 2.97 | ± 0.11 | 3.01 | ± 0.07 |  | 2.43 | | ± 0.07 | 2.61 | ± 0.10 |
| HDL Cholesterol (mmol/L) | 2.02 | ± 0.07 | 2.04 | ± 0.05 |  | 1.59 | | ± 0.04 | 1.71 | ± 0.07 |
| Glucose (mmol/L) | 9.19 | ± 0.39 | 9.64 | ± 0.29 |  | 8.63 | | ± 0.24 | 8.90 | ± 0.27 |
| Creatinine (µmol/L) | 27.8 | ± 1.0 | 27.3 | ± 0.5 |  | 27.7 | | ± 0.5 | 27.7 | ± 0.6 |
| Urea (mmol/L) | 10.23 | ± 0.58 | 9.40 | ± 0.23 |  | 9.35 | | ± 0.46 * | 7.46 | ± 0.33 |
| Total bilirubin (µmol/L) | 2.17 | ± 0.17 | 2.00 | ± 0.13 |  | 1.83 | | ± 0.15 | 1.86 | ± 0.15 |
| TAS ^5^ (mmol/L) | 1.63 | ± 0.11 | 1.83 | ± 0.06 |  | 1.63 | | ± 0.08 | 1.63 | ± 0.10 |
| Total proteins (g/L) | 54.6 | ± 0.9 | 54.7 | ± 0.9 |  | 53.6 | | ± 0.4 * | 55.6 | ± 0.9 |

^1^: *n* = 12; ^2^: *n* = 23; ^3^: *n* = 23; ^4^: *n* = 14; ^5^ TAS: total antioxidant status.

**P* < 0.05 using Student’s *t*-test.
