# Supplementary material for: Brain IGF-1 Receptors Control Mammalian Growth and Lifespan through a Neuroendocrine Mechanism
Source: PLoS Biol. 2008 Oct 28;6(10):e254. doi: 10.1371/journal.pbio.0060254 (PMC2573928; doi:10.1371/journal.pbio.0060254)
Supplement: Text S1 — (74 KB DOC) [file pbio.0060254.sd001.doc]

SUPPORTING TEXT

SUPPLEMENTARY RESULTS

**Early dietary restriction mimics the bIGF1RKO^+/-^ phenotype**

We showed here that reduced IGF signaling in the developing brain leads to marked GH and IGF-I deficiency, raising the question whether such mechanism could be physiologically relevant. Circulating IGF-I is a reliable nutritional marker during early life [1,38], and we hypothesized that decreased IGF-I secondary to low nutrition could also specifically down regulate growth by inhibiting the development of the somatotropic axis. This in turn would contribute to adjusting individual growth to available nutritional resources. To test this hypothesis, we restricted nutrition in wild type mice during the first two weeks of life only. Restricted mice promptly exhibited progressive growth retardation (-15% body weight on day 20, *P* < 0.001, Supplementary Fig. 5A; body length, 76.7 ± 0.7 versus 80.3 ± 0.5 mm, *P* < 0.001; *n* = 15). Moreover, although restriction was limited to only the first two weeks of life, these mice did not catch up with normal growth afterwards, and presented as adults still 6% less body weight compared to normal (*P* < 0.001). Glycemia was reduced at 10 days of age, most likely secondary to food restriction, but normalized at 20 days, under *ad libitum* feeding (not shown). Similarly, leptinemia (reflecting adiposity) was halved in restricted mice at 10 days (Supplementary Fig. 5B left), but close to normal at 20 days. In addition, in restricted mice, GHRH mRNA was low as early as day 10 (Supplementary Fig. 5B right), SRIH was unchanged (not shown), and pituitary GH content was low at day 20 (Supplementary Fig. 5C left panel), all of which was highly reminiscent of the bIGF1RKO^+/-^ phenotype. Finally, circulating IGF-I levels were diminished at day 10, as a direct result of restricted nutrition, and remained low at day 20 despite *ad libitum* feeding (Supplementary Fig. 5C right), likely attributable to the lasting GH deficiency. Importantly, differences in circulating IGF-I did continue into late adulthood: we found significantly decreased IGF-I at 12 months in the previously restricted mice, compared to normal mice (not shown).

SUPPLEMENTARY DISCUSSION

**Genetic background and control populations**

Genetic background has significant impact on developmental phenotype, adult physiology and lifespan, and variations in background between mutant and controls may produce bias. Moreover, mouse F1 hybrids are known to be naturally long-lived, due to so-called *hybrid vigor*. For these reasons, we produced bIGF1RKO^+/-^ mice and their controls as F1 hybrids with identical 129/B6 background (see methods section for details). To further validate this strategy, we compared survival data of our controls with a panel of normal mouse strains, including 8 different F1 hybrids produced from major inbred strains [6]. We found that the mean lifespan of our controls (836 d) compared well with the hybrid populations studied by Smith et al. (mean lifespan 822 d). This was also true when separating into sexes: The mean lifespan of control males from our study was very similar to other F1 hybrids (853 versus 850 d), while our female controls lived even slightly longer than comparable hybrid strains (821 versus 793 d) [6]. Since the bIGF1RKO^+/-^ mutation shifted the survival curves of males and females in a very similar fashion, we performed Kaplan-Meier survival analysis for both sexes separately and also with the combined male and female populations. Moreover, the control groups of an independent lifespan experiment, with identical genetic background and housing conditions, presented the same mean lifespan (823 ± 22 d, -1.6%, *P* = 0.68) and highly similar survival curves (not shown) compared to bIGF1RKO^+/-^ controls.

SUPPLEMENTARY METHODS

**Genotyping**. Mice were genotyped by multiplex PCR using DNA from skin biopsies. Primers 5’-CCATGGGTGTTAAATGTAATGGC-3’, 5’-ATGAATGCTGGTGAGGGTTGTCTT-3’ and 5’-ATCTTGGAGTGGTGGGTCTGTTTC-3’ were simultaneously used to amplify DNA fragments from wild-type (256 bp), floxed (312 bp), and Cre-lox recombined (204 bp) *Igf1r* alleles. The transgene *NesCre* was detected using Cre cDNA primers 5’-CCTGGAAAATGCTTCTGTCCG-3’ and 5’-CAGGGTGTTATAAGCAATCCC-3’ (392 bp), and Gabra1 primers 5'-AACACACACTGGCAGGACTGGCTAGG-3' and 5'-CAATGGTAGGCTCACTCTGGGAGATGATA-3' as positive control.

**Body composition**. We dissected 10-month-old males and females to determine organ and tissue weight. We measured naso-anal body length and sampled blood by cardiac puncture under pentobarbital anesthesia. The brain was removed and the pituitary gland prepared, immediately weighed and frozen. Limb muscles were sectioned at their proximal insertions, limbs removed at their proximal joints, and feet sectioned (bones accounted for about 5% of limb weight; the rest being muscle). The major abdominal and thoracic organs were prepared. Coagulated blood was removed from the heart before weighing. Weight of the principal adipose tissue (AT) compartments was determined. Gonadal (epididymal/uterine), mesenteric (peritoneal) and perirenal (retroperitoneal) fat pads represented the visceral (intra-abdominal) AT. The inguinal and dorsolumbar fat pads (on proximal hind legs and lower trunk) and the interscapular fat pads (on back and upper trunk) represented the subcutaneous AT. Any connective tissue removed during the dissection was added to the carcass, which comprised essentially the skeleton and connective tissue from the body and the head.

**Southern analysis**. Genomic DNA was prepared from 50-100 mg tissue and Southern analysis performed as described [7]. DNA (10 µg) from each tissue was digested with *Hin*cII and I-*Sce*I, or *Hin*cII alone, fractionated on 1% agarose gel, transferred to Hybond-N+ membrane, UV fixed and probed with a radiolabeled (Rediprime) 0.75 kb fragment of the *Igf1r* gene.

**Northern** **analysis***.* A Spi2.1 cDNA probe [8,9], kindly provided by A. Le Cam, was radiolabeled using Rediprime. Total RNA (10 µg per sample) were fractionated on 1% agarose gel containing formaldehyde in 1 x MOPS buffer. RNA was blotted to Hybond-C and UV fixed (Stratagene). Prehybridized (3 h) and hybridized overnight at 42 °C, the membranes were washed in 0.1 x SSC, 0.1% SDS at 65 °C final stringency, and placed against X-ray film with amplifying screens at -80 °C for 2 weeks.

***In vitro* ligand binding assay** **and western ligand blot**. Recombinant human IGF-I (rhIGF-I) and -II were labeled with ^125^I. *In vitro* ligand binding assay was described elsewhere [7]. For IGFBP western ligand blotting, 3 µL plasma were subjected to 12.5% non-reducing PAGE. Proteins were electrotransferred to nitrocellulose membrane and incubated with ^125^I-IGF-I and -II (500,000 cpm each). Blots were quantified using a Molecular Dynamics STORM 850 PhosphorImager and ImageQuant 5.0 software. A pooled sample was included on each gel for comparison.

**Behavior**, *Actimetry*. Circadian locomotor activity was measured using a computer-controlled photoelectric actimeter. For subsequent tests (see below), we used computer video-tracking to capture and quantify the movements of the animals over time.
*Open-field* and *novel object test*: Mice were placed in a 45 x 45 cm arena, in which visits to the central zone of 27 cm diameter reflect the conflict between exploration and anxiety. Entries into, time spent and distance traveled in the central zone were recorded over 30 minutes. Immediately afterwards, we assessed explorative behavior independently of anxiety by placing a new object in the open-field arena, and measuring the same set of parameters for another 30 minutes.

*Y-maze*. Short-term spatial memory was tested by placing mice individually for 3 min in a point-symmetric Y-shaped maze (25 x 7 x 14 cm each arm) with one closed arm. Distant visual cues above the maze allowed spatial orientation. Two hours later, animals were placed again in the maze for 5 min, with the third arm open. Number of visits and time spent in the different arms were measured.

O*-maze*. Anxiety was measured using a circular platform with an outer diameter of 46 cm, and 7 cm wide/14 cm high lateral protections covering two 90° sectors on opposite sides. Three types of zone were defined on the platform: two opposite 50° sectors in the centers of the protected parts of the platform; two opposite 70° sectors in the centers of the open parts of the platform; four intermediate 30° sectors, each separating the other two types of sector. Over a 10-minute period, we analyzed the number of entries into, time spent and distance traveled in the unprotected anxiogenic areas of the platform.

SUPPLEMENTARY NOTES

References in Supporting Text

1. Thissen JP, Ketelslegers JM, Underwood LE (1994) Nutritional regulation of the insulin-like growth factors. Endocr Rev 15: 80-101.

2. Voikar V, Koks S, Vasar E, Rauvala H (2001) Strain and gender differences in the behavior of mouse lines commonly used in transgenic studies. Physiol Behav 72: 271-281.

3. Freeman M (2000) Feedback control of intercellular signalling in development. Nature 408: 313-319.

4. Yakar S, Liu JL, Stannard B, Butler A, Accili D, Sauer B, LeRoith D (1999) Normal growth and development in the absence of hepatic insulin-like growth factor I. Proc Natl Acad Sci U S A 96: 7324-7329.

5. Giustina A, Veldhuis JD (1998) Pathophysiology of the neuroregulation of growth hormone secretion in experimental animals and the human. Endocr Rev 19: 717–797.

6. Smith GS, Walford RL, Mickey MR (1973) Lifespan and incidence of cancer and other diseases in selected long-lived inbred mice and their F 1 hybrids. J Natl Cancer Inst 50: 1195-1213.

7. Holzenberger M, Leneuve P, Hamard G, Ducos B, Perin L, Binoux M, Le Bouc Y (2000) A targeted partial invalidation of the IGF-I receptor gene in mice causes a postnatal growth deficit. Endocrinology 141: 2557-2566.

8. Pages G, Rouayrenc JF, Le Cam G, Mariller M, Le Cam A (1990) Molecular characterization of three rat liver serine-protease inhibitors affected by inflammation and hypophysectomy. Protein and mRNA analysis and cDNA cloning. Eur J Biochem 190: 385-391.

9. Shayiq RM, Avadhani NG (1992) Sequence complementarity between the 5’-terminal regions of mRNAs for rat mitochondrial cytochrome P-450c27/25 and a growth hormone-inducible serine protease inhibitor. A possible gene overlap. J Biol Chem 265: 2421-2428.
